# Pedicled omental split skin graft: A novel method for reconstruction of full-thickness abdominal wall defect

**DOI:** 10.4103/0971-9261.72440

**Published:** 2010

**Authors:** Anindya Chattopadhyay, Sumitra Kumar Biswas, Meghnad Dutta

**Affiliations:** Department of Pediatric Surgery, Dr. BC Roy Memorial Hospital for Children, 111, Narkeldanga Main Road, Kolkata, India

**Keywords:** Abdominal wall defect, aggressive fibromatosis, desmoid tumor, pedicled omental split skin graft

## Abstract

Although rare in children, aggressive fibromatosis or desmoid tumors require wide surgical excision for durable relief. The resultant defect poses many challenges for reconstruction. The authors report reconstruction of such a wound using a pedicled omental split skin graft, which resulted from the excision of a locally recurrent desmoid tumor.

## INTRODUCTION

Aggressive fibromatosis or desmoid tumor is a rare soft tissue tumor in the pediatric age group.[1] Arising from connective tissue and its overlying fascia, the tumor has a predilection for certain sites such as the shoulder, chest wall, thigh or head-neck.[2] Although benign, the lesion has a high local recurrence rate after excision,[3] and excision with clear surgical margins provide the best hope for a cure.[1]

The authors were involved in the management of a large recurrent chest and abdominal wall desmoid, whose surgical extirpation led to the creation of a full thickness abdominal wall defect, which was reconstructed using the pedicled omentoplasty and split skin graft (POSSG) technique.

## CASE REPORT

A 12-year-old female presented with a rapidly increasing mass located over the lower left chest wall and the abdomen. The mass had first been noted in the year 2000, and was confined to the chest wall, and local excision had been done in the same year. Histology had revealed aggressive firbomatoisis and the child was on follow up for a year, after which she dropped out.

In 6 months, the mass made a reappearance in the scar tissue of the previous surgery. She underwent a second excisional surgery in 2003, when the tumor involved the upper part of both recti and excision was accomplished with excision of the anterior part 9^th^ and 10^th^ ribs. The defect was repaired by inserting a prolene mesh, and the skin was closed primarily.

After 6 months, the tumor recurred and the parents started traditional treatment and did not report for follow-up. The mass grew extremely slowly over the next 2 1/2 years, but since then had grown aggressively to reach large dimensions.

**Figure 1:**
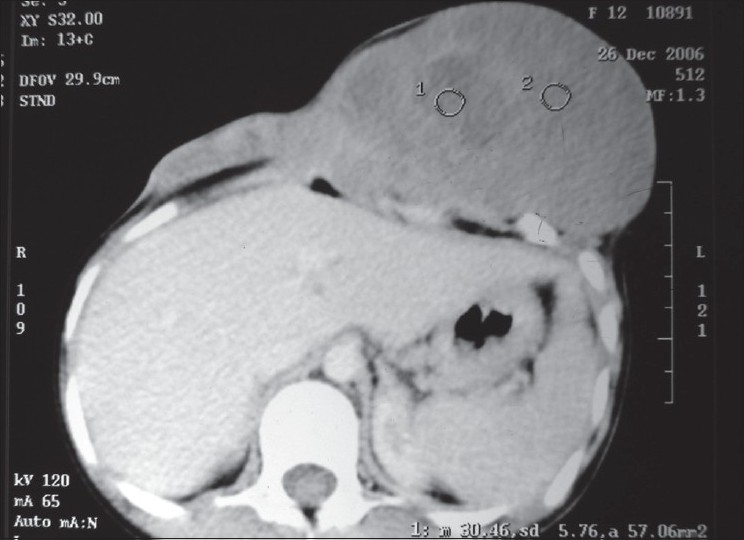
Computed tomography scan of the abdomen showing the large tumor arising from the anterior abdominal wall

General physical examination was unremarkable. Local examination revealed a 15 cm × 12 cm exophytic nodular mass arising from the lower left chest wall and the contiguous anterior abdominal wall. The mass was hard in consistency, crossing the midline, with scars of the previous surgery visible over the mass. Investigations revealed a normal hemogram, renal functions and liver function. Chest radiograph was normal and a computed tomogram (CT) scan revealed that the mass was arising from the lower chest wall and abdominal wall, and was free from the underlying viscera, including the liver [Figure 1]. With a diagnosis of recurrent desmoid tumor, excisional surgery was planned. Reconstruction of the defect that would result could have been done with a musculofascial flap. However, in view of the two earlier recurrences, the location and size of the defect, the possible need for a microvascular anastomosis and with the history of drop out from follow-up, we decided to use a prosthetic mesh surfaced with a skin graft for reconstruction.

Excision was accomplished with resections of the anterior portions of the 8^th^ and 9^th^ ribs. The mesh placed previously was removed. The diaphragm was sutured to the 7^th^ rib, closing the thoracic cavity. This left a full-thickness defect on the anterior abdominal wall measuring 14 cm × 12 cm. The omentum was detached from the transverse colon and the right gastroepiploic artery was divided. The omentum was then detached from the greater curvature, keeping the epiploic arcade intact. A prolene mesh was then sutured to the muscles in the margin of the wound, leaving a small gap superomedially through which the omentum was brought out and spread over the surface of the mesh. The omentum was loosely tacked to the mesh and skin margins and covered with Vaseline gauze and a dressing was applied.

On the 7^th^ postoperative day, she was returned to the operation theater and split skin grafts were taken from the thigh and placed on the granulating surface of the omentum. She recovered without any complications and was discharged after 10 days with a well-taken skin graft covering the mesh. Histology revealed a desmoid tumor with clear surgical margins.

**Figure 2:**
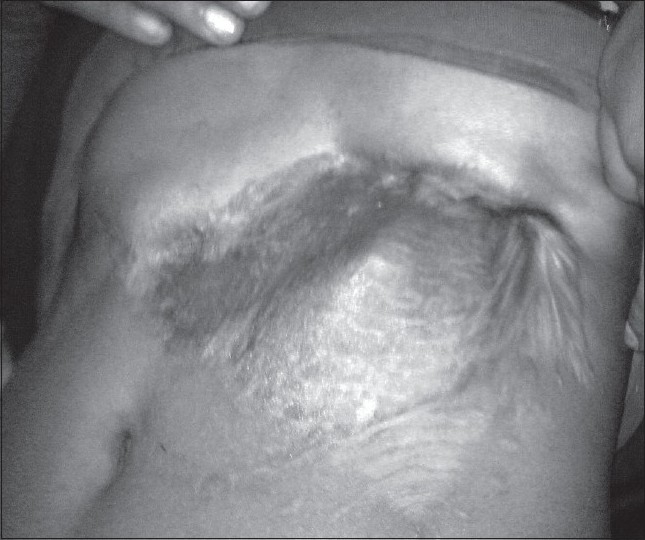
Patient 2 years after surgery. Note the lack of any abdominal wall weakness

On follow-up, she had a small nonhealing discharging sinus on the superomedial aspect of the wound that responded to local probing and removal of a suture. She has been on follow-up for 3 years without any evidence of local recurrence or ventral hernia and she is pleased with the outcome [Figure 2].

## DISCUSSION

Aggressive fibromatosis or desmoid tumors are soft tissue tumors that are rare in the pediatric age group.[1] Consisting of elongated spindle cells and arising from muscles or their aponeurosis, these tumors never metastasize but cause significant morbidity from their propensity for local invasion and recurrence, which can approach 50%.[3] These tumors are slow growing, and show a predilection for certain sites such as the shoulder girdle, chest wall, back and thigh, with a significant percentage occurring intraabdominally.[2]

Surgical excision without damage to vital neurovascular structures remains the cornerstone of therapy.[1] Patients who achieve clear margins microscopically have low recurrence rates compared with those with positive or uncertain margins.[4] In an effort to reduce recurrences, radiotherapy (5000–6000 Cgy) or chemotherapy in the form of Vincristine, Actinomycin-D and Cyclophosphamide (VAC) has been tried with some success,[2–4] but with the attendant concern for the effects on growth and potential second malignancies.

Adequate excision of these tumors proves a challenge for reconstructing the resultant defect, especially in areas such as the chest wall and upper abdomen. The goals of reconstruction are to provide optimum structural support with stable soft tissue and good aesthetic results.[5] Division of the abdomen into various zones with appropriate myocutaneous flaps for reconstruction of defects has been proposed.[6,7] Lateral and upper abdominal defects may be reconstructed with latissimus dorsi myocutaneous flap and lower defects with a tensor fascia lata myocutaneous flap.[6] The versatility of the flaps may be improved by using tissue expanders[7] or expanding the vascular territory by using microvascular anastomosis.[5] These flaps have the potential for a better cosmetic result, but carry the attendant risks of flap loss and recurrence beneath the flap, which may be difficult to detect. After excising the tumor with a margin, we were faced with a large musculofascial and skin defect that was successfully reconstructed using a mesh surfaced with omentum and covered with a split skin graft. The POSSG has been previously used in reconstruction of chest and abdominal wall defects with reliable results,[8–10] but this is probably the first reported pediatric case.

The procedure as described above is technically simple and requires no specialized instrumentation or microvascular anastomosis and can thus be used in centers with limited resources.

## References

[1] Buitendijk S, van de Ven CP, Dumans TG, den Hollander JC, Nowak PJ, Tissing WJ (2005). Pediatric aggressive fibromatosis. Cancer.

[2] Ayala AG, Ro JY, Goepfert H, Kangir A, Khorsand J, Flake G (1986). Desmoid fibromatosis: a clinicopathologic study of 25 children. Semin Diagn Pathol.

[3] Rao BN, Horowitz ME, Parham DM, Etcubanas EE, Fleming ID, Pratt CB (1987). Challenges in the treatment of childhood fibromatosis. Arch Surg.

[4] Faulkner LB, Hajdu SI, Kher U, La Quaglia M, Exelby PR, Heller G (1995). Pediatric desmoid tumor; retrospective analysis of 63 cases. J Clin Oncol.

[5] Dorai AA, Halim AS (2007). Extended double pedicle free tensor fascia latae myocutaneous flap for abdominal wall reconstruction. Sing Med J.

[6] Sharma RK, Singh G, Naidu PM (1998). Abdominal wall defects: anatomic classification and a scheme for management. Ann Plastic Surg.

[7] Mathes SJ, Steinwald PM, Foster RD, Hoffman WY, Anthony JP (2000). Complex abdominal wall reconstruction: a comparison of flap and mesh closure. Ann Surg.

[8] Blom WF, Koops HS, Vermey A, Oldhoff J (1982). Abdominal wall resection and reconstruction with the aid of Marlex mesh. Br J Surg.

[9] Contant CM, van Geel AN, van der Holt B, Wiggers T (1996). The pedicled omentoplasty and split skin graft (POSSG) for reconstruction of large chest wall defects. A validity study of 34 patients. Eur J Surg Oncol.

[10] El-Muttardi N, Lancaster K, Ng R, Mercer D (2005). The sandwich omental flap for abdominal wall defect reconstruction. Br J Plast Surg.

